# Saliency Mapping Enhanced by Structure Tensor

**DOI:** 10.1155/2015/875735

**Published:** 2015-12-14

**Authors:** Zhiyong He, Xin Chen, Lining Sun

**Affiliations:** ^1^School of Mechanical and Electric Engineering, Soochow University, Suzhou 215021, China; ^2^NovuMind Inc., Santa Clara, CA 95131, USA

## Abstract

We propose a novel efficient algorithm for computing visual saliency, which is based on the computation architecture of Itti model. As one of well-known bottom-up visual saliency models, Itti method evaluates three low-level features, color, intensity, and orientation, and then generates multiscale activation maps. Finally, a saliency map is aggregated with multiscale fusion. In our method, the orientation feature is replaced by edge and corner features extracted by a linear structure tensor. Following it, these features are used to generate contour activation map, and then all activation maps are directly combined into a saliency map. Compared to Itti method, our method is more computationally efficient because structure tensor is more computationally efficient than Gabor filter that is used to compute the orientation feature and our aggregation is a direct method instead of the multiscale operator. Experiments on Bruce's dataset show that our method is a strong contender for the state of the art.

## 1. Introduction

*Visual saliency* (also called* visual salience*) refers to the quality or state by which information stands out relative to its neighbors and often attracts human attention [1]. In the subsequent stages, the salient images are preferentially taken as inputs instead of the whole image. As a consequence, visual saliency has been widely applied to various computer vision tasks such as segmentation [2, 3], image retargeting [4–6], object detection [7, 8], image collection [9], and object recognition [10].

Koch and Ullman introduced a basic biologically inspired architecture of visual saliency, referred to as Koch and Ullman model [11]. Then Itti et al. presented a computational architecture to implement and verify the Koch and Ullman model [12]. As summarized in [13], most of the implementation techniques of the visual saliency models generally have three stages: (1)* extraction:* extracting low-level features at locations over the image plane, (2)* activation:* forming activation maps from the features, and (3)* normalization/combination:* normalizing the activation maps and combining them into a single saliency map.

For Itti method, the objective of the first stage is to extract three low-level features, intensity, color, and orientation, and followed by using Difference of Gaussian (DOG) to form total of forty activation maps. Finally, a linear operator is employed to normalize these maps, in which the most salient location is selected by the winner-take-all neural network to generate a saliency map. However, results of Itti method are sometimes blurry and prefer small and local features, which are less useful for some further computer vision applications such as object segmentation and detection.

Despite many advances of the visual saliency made in recent years, the various evaluation results in [14] indicate that there are still some questions about the mechanism of visual saliency. In addition to a motivation of investigation on some questions such as low-level features and the combination of activation maps, in this paper, we also focus on the performance of algorithm and whether the saliency results can greatly benefit computer vision applications.

Results from some recent research works have shown that features of edge and corner also play important roles in visual saliency [13, 15, 16]. In our study, we also note that orientation features are less likely to win in the combination of activation maps. Moreover, Gabor filters used for orientation extraction are computationally expensive.

**Figure 1:**
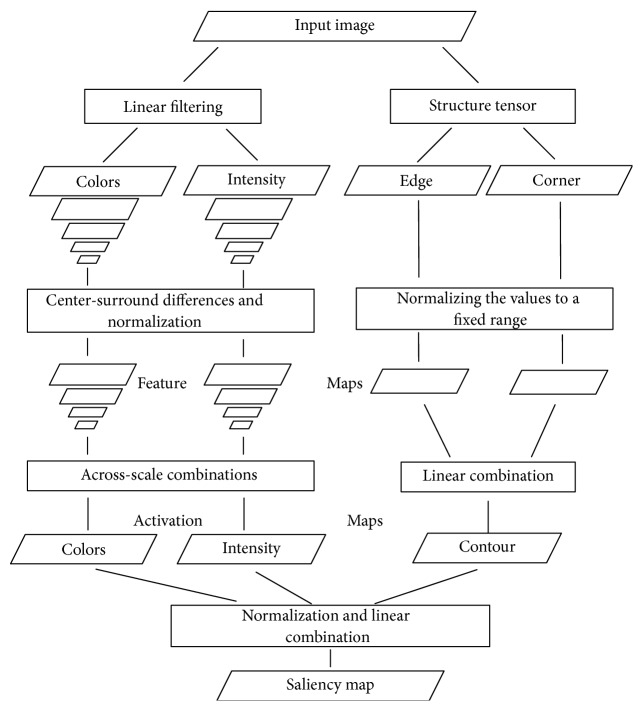
General architecture of our method. In our method, we call the activation map generated by edge and corner features contour activation map. The final saliency map combined these activation maps into ST saliency map.

We therefore propose an efficient method to compute saliency map, referred to as* structure tensor* (ST) saliency map. The computational architecture of our method is shown in Figure 1 and is the same as Itti method in feature extraction and activation maps generation for intensity and color features. The features of edge and corner are extracted by structure tensor and directly combined into an activation map, called contour map. After obtaining three activation maps, we use linear combination to aggregate activation maps to a saliency map instead of multiscale combination and winner-take-all rule.

This paper makes two major contributions as follows:

1. We propose a novel efficient algorithm to calculate the saliency map. Compared to other methods performed on a challenging dataset, besides the best performance achieved, the results of our method obtain sharper boundaries which are useful in some further applications such as object segmentation and detection.
2. Our work has shown that edge and corner are two important low-level features in saliency generation.

The paper is organized as follows.  Section 2 briefly reviews the state-of-the-art methods with particular emphasis on saliency algorithms related to Itti method, and Section 3 introduces some backgrounds of structure tensor and formally describes our algorithm of saliency map computation. In Section 4, we present our experimental results and quantitative evaluations on a challenging dataset and discuss them. This paper closes with a conclusion of our work in Section 5.

## 2. Related Work

Visual saliency methods are generally categorized into biologically inspired methods and computationally oriented methods. There is an extensive literature on the areas, but here we mention just a few relevant papers. Some surveys are found in [17–19], and some recent progress is reported in [20].

Koch and Ullman [11] proposed a basic architecture of biologically inspired methods and defined a saliency map as a topographic map that represents conspicuousness of scene locations. Their work also introduced a winner-take-all neural network that selects the most salient location and employs an inhibition of return mechanism to allow the focus of attention to shift to the next most salient location. Then Itti et al. presented a computational model to implement and verify Koch and Ullman model. Since then, the works related to the saliency map have quickly become one of the hot research fields.

Itti method employs a Difference of Gaussian (DOG) operator to evaluate color, intensity, and orientation features to generate total of forty activation maps and across-scale-combines these maps into a saliency map. Besides the expensive computation, one big problem of Itti method is that the results are sometimes blurry and prefers small purely local features. On the other hand, many algorithms of computer vision need input features related to contours because they require the distinct boundary information. Recently, some methods have been proposed to obtain sharp edges, for example, local dissimilarities at the pixel level [21], multiscale DOG [22], and histogram analysis [15]. However, the common problem of these methods is that they are more sensitive to the noises.

As mentioned in the previous section, improving on Itti method, we propose an efficient algorithm for calculating the saliency map, and the computational architecture of our method is shown in Figure 1. The computational architecture of our method is similar to Itti method, and our method evaluates intensity, color, edge, and corner features instead of intensity, color, and orientation features. The structure tensor is used to extract the features of the edge and corner. In the final step, we use the linear combination to generate a saliency map instead of the winner-take-all rule of Itti method.

## 3. The Proposed Saliency Model

In this section, we briefly introduce the background of structure tensor and formally describe our algorithm.

### 3.1. Introduction to Structure Tensor

In mathematics, structure tensor is a matrix representation of partial derivative information. In the field of image processing and computer vision, it typically represents the gradient or edge information and has a more powerful description of local patterns as opposed to the directional derivative through its coherence measure [23, 24].

There are two categories of structure tensor: linear structure tensor and nonlinear structure tensor. Compared to the nonlinear structure tensor, the linear structure tensor is fast and easy to implement with Fast Fourier Transform (FFT). We therefore select the linear structure tensor to extract the features of edges and corners.

Given an image *I*(*x*, *y*), if pixel (*x*, *y*) translates to (*x* + Δ*x*, *y* + Δ*y*), the energy *E* is defined as

(1) $$\begin{array}{c} E=\underset{\left(u,v\right)\in W\left(x,y\right)}{\sum }w\left(\;u,v\;\right){\left(\;I\left(\;u,v\;\right)-I\left(\;u+\Delta x,v+\Delta y\;\right)\;\right)}^{2}, \end{array}$$

where *W*(*x*, *y*) is a window at center point (*x*, *y*) and *w*(*u*, *v*) is a weight function at pixel (*x*, *y*). In the rest of this paper, ∑_(*u*,*v*)∈*W*(*x*,*y*)_ *w*(*u*, *v*) is simply written as ∑_*w*_.

It is approximated by a first-order Taylor series:

(2) Iu+Δx,v+ΔyIu,v+∂I∂xu,vΔx+∂I∂yu,vΔy=Iu,v+∂I∂xu,v,∂I∂yu,vΔxΔy.

Hence, (1) can be rewritten as

(3) E∑wIu,v−Iu+Δx,v+Δy2≈Δx,ΔyTΔxΔy,

where matrix **T** is

(4) T∑w∂I∂x2∂I∂x·∂I∂y∂I∂x·∂I∂y∂I∂y2=∑w∂I∂x2∑w∂I∂x·∂I∂y∑w∂I∂x·∂I∂y∑w∂I∂y2.

Matrix **T** is a structure tensor, which is also considered as a covariance matrix.

**Figure 2:**
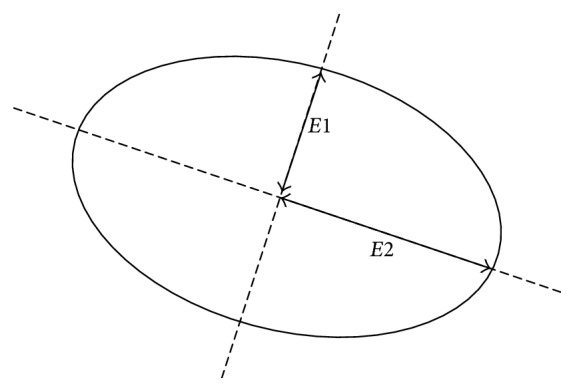
The relation between ellipse and structure tensor.

We also consider (3) as an approximation of a binomial function, and from a view of geometry, a binomial function is an ellipse where short axis and long axis are represented as eigenvalues *E*1 and *E*2, respectively. The direction of ellipse is determined by the eigenvectors. As shown in Figure 2, the equation of ellipse is written as

(5) $$\begin{array}{c} \left[\;\Delta x,\Delta y\;\right]T\left[\begin{array}{c} \Delta x\\ \Delta y \end{array}\right]=1. \end{array}$$

Based on (4), some types of structure tensor have been constructed. In our work, we use a linear structure tensor to analyze the input image, and it is defined as

(6) $$\begin{array}{c} J_{\sigma }=\overset{3}{\underset{i=1}{\sum }}\left[\begin{array}{cc} K_{\sigma }*{\left(\frac{\partial I_{i}}{\partial x}\right)}^{2} & K_{\sigma }*\left(\frac{\partial I_{i}}{\partial x}\cdot \frac{\partial I_{i}}{\partial y}\right)\\ K_{\sigma }*\left(\frac{\partial I_{i}}{\partial x}\cdot \frac{\partial I_{i}}{\partial y}\right) & K_{\sigma }*{\left(\frac{\partial I_{i}}{\partial y}\right)}^{2} \end{array}\right], \end{array}$$

where *K*_*σ*_ is a Gaussian kernel with variance *σ* and *∗* is a convolution operator. The parameter *i* is the image channel number.

For any kind of structure tensor, we use $\left[\begin{array}{cc} G & F\\ F & H \end{array}\right]$ to simply represent matrix **T** of (4). Then the two eigenvalues are calculated as

(7) λ1=G+H+G−H2+4F22,

(8) λ2=G+H−G−H2+4F22.

### 3.2. Contour Activation Map

As shown in Figure 1, we calculate the activation maps of color and intensity with Itti method, and the contour activation map is detailed in Algorithm 1.

For computation of **A** and **B**, we do not need to compute *λ*_1_ and *λ*_2_ with (7) and (8) and add and subtract these values to calculate **A** and **B**. We directly compute them as

(9) λ1−λ2=G−H2+4F2,λ1+λ2=G+H.

In the final step, we combine feature maps into a contour activation map **C** **T** as follows:

(10) $$\begin{array}{c} \text{CT}=\frac{1}{2}\left(\;N\left(\;A\;\right)+N\left(\;B\;\right)\;\right), \end{array}$$

where *N*(*A*) is the normalized edge feature map and *N*(*B*) is the normalized corner feature map.

### 3.3. ST Saliency Map Generation

We assume that all features equally contribute to the ST saliency map generation. After obtaining the contour activation map, the intensity activation map, and the color activation map, we combine them into a saliency map as follows:

(11) $$\begin{array}{c} S=\frac{1}{3}\left(\;⨁\left(\;\text{CL}\;\right)+⨁\left(\;I\;\right)+⨁\left(\;\text{CT}\;\right)\;\right), \end{array}$$

where ⨁ is a normalization operation which is defined in [12], **C** **L** is the color activation map, **I** is the intensity activation map, and **C** **T** is the contour activation map.

**Figure 3:**
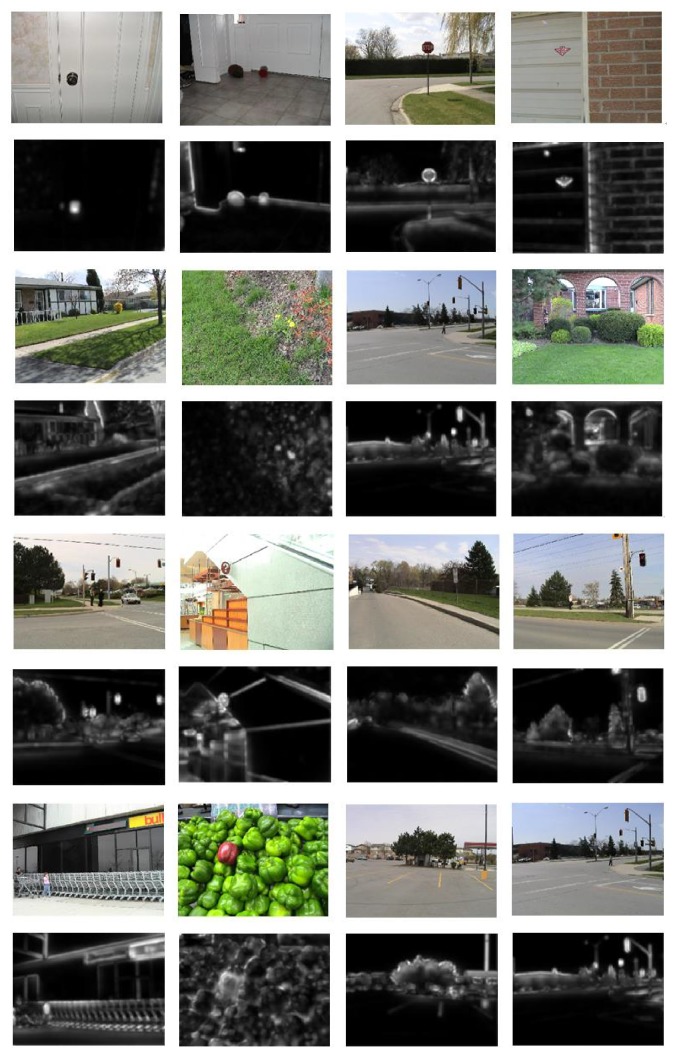
Saliency maps of our method. The odd row is the input images from Bruce dataset, and the even row is the saliency generated by our method. Obviously, the saliency maps of our method have sharp edges.

Some saliency maps of our method are shown in Figure 3 and these maps have distinct boundaries.

## 4. Experimental Results

In this section, we present subjective evaluation and quantitative analysis of our method and some state-of-the-art methods and analysis of performance of these methods.

### 4.1. Saliency Maps

**Figure 4:**
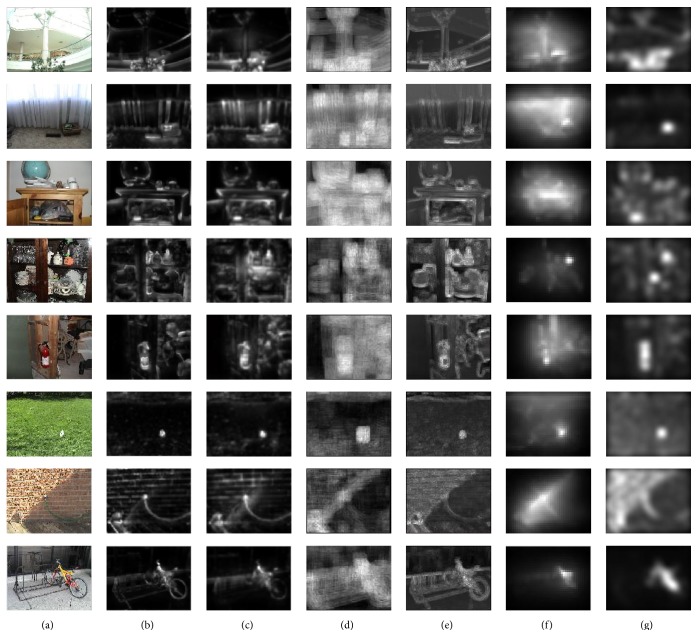
Saliency maps on the Bruce dataset. (a) Input image, (b) our method, (c) Itti method, (d) AIM method, (e) DVA method, (f) GBVS method, and (g) IS method using LAB color space. Since our method includes the edge and corner information, saliency maps of our method have sharp edges that are useful for the further steps in some computer vision tasks.

We compared saliency maps of our method with saliency maps of some state-of-the-art methods including Itti method [12], Attention Based on Information Maximization (AIM) method [25, 26], Dynamic Visual Attention (DVA) method [27], Graphic-Based Visual Saliency (GBVS) method [13], and Image Signature (IS) method [28]. The MATLAB implementation of these methods is based on the codes on the authors' websites. Saliency maps are shown in Figure 4.

### 4.2. Analysis of Performance

We evaluated our method on Bruce dataset containing 120 natural images with eye fixation ground truth data. In Bruce dataset, the size of all images is 681 × 511. Some of methods are sensitive to different sizes of the input image. As a consequence, in order to fairly evaluate results of different methods, we resize the input images to the same size (170 × 128) for each method.

**Figure 5:**
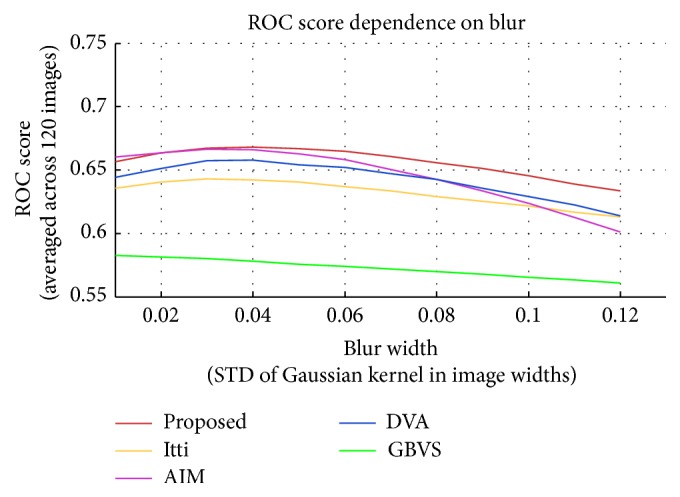
Comparison of the ROC AUC scores of all methods. Our method achieves the best ROC AUC score.

Results from perceptual research works [29, 30] have found that human fixations have strong center bias which may affect the performance of a saliency algorithm. To remove this center bias, following the procedure of Tatler et al.'s work [29], Hou et al. [28] introduced ROC Area Under the Curve (AUC) score to quantitatively evaluate the performance of different algorithms. Good results should maximize the ROC AUC score. To compare the ROC AUC scores, we follow the computation method provided by [28], but the size (170 × 128) is different with two input image sizes used in [28]. Comparison of the ROC AUC scores is shown in Figure 5.

We conducted our tests on a laptop with Intel Dual-Core i5-4210U 1.7 GHz CPU and 4 G RAM memory. All codes were written in MATLAB.

**Figure 6:**
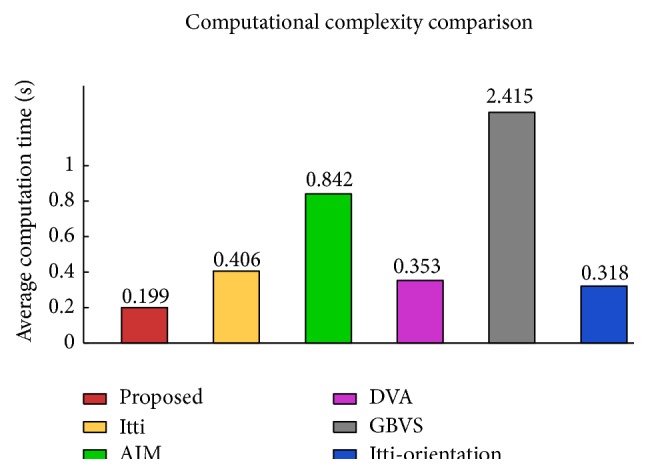
Results of the performance of these different methods. Time measurements are given in seconds. The results are the average times of 120 images of Bruce dataset.

The execution times of the methods are summarized in Figure 6, in which the time is an average time of 120 images. The figure shows that our method is about twice as fast as Itti method and outperforms other state-of-the-art methods. The reason lies in two parts. First, structure tensor is an efficient algorithm of feature extraction. Second, we directly aggregate three activation maps into a saliency map. It is obvious that the performance will increase greatly if our method is implemented by C/C++, and it should satisfy most of the real time applications.

## 5. Conclusion

In this paper we have proposed an efficient algorithm for computing the saliency map, which has a distinct boundary that contributes to further computer vision applications such as segmentation and detection. The computational architecture of our method is close to Itti method, but we have made two improvements in low-level features extraction and combination of activation maps. Since features of edge and corner are important cues in visual saliency, we use a linear structure tensor to extract these features. The reason that our algorithm is efficient lies in the following: (1) linear structure tensor is an efficient feature extraction algorithm and (2) our linear combination method is fast. On the basis of experimental results on Bruce dataset, our method has shown that some computer vision tasks, in particular real time applications, can benefit from our method as a preprocessing step.

## Figures and Tables

**Algorithm 1:**
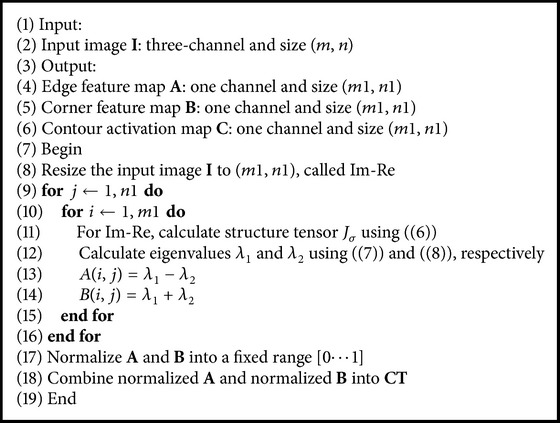
Algorithm of contour activation map.
